# The Kink Turn, a Key Architectural Element in RNA Structure

**DOI:** 10.1016/j.jmb.2015.09.026

**Published:** 2016-02-27

**Authors:** Lin Huang, David M.J. Lilley

**Affiliations:** Cancer Research UK Nucleic Acid Structure Research Group, MSI/WTB Complex, The University of Dundee, Dow Street, Dundee DD1 5EH, United Kingdom

**Keywords:** k-turn, RNA folding, metal ions, tertiary interactions, RNA–protein interaction

## Abstract

Kink turns (k-turns) are widespread structural elements that introduce an axial bend into duplex RNA with an included angle of 50°. These mediate key tertiary interactions and bind specific proteins including members of the L7Ae family. The standard k-turn comprises a three-nucleotide bulge followed by G·A and A·G pairs. The RNA kinks by an association of the two minor grooves, stabilized by the formation of a number of key cross-strand hydrogen bonds mostly involving the adenine bases of the G·A and A·G pairs. The k-turns may be divided into two conformational classes, depending on the receptor for one of these hydrogen bonds. k-turns become folded by one of three different processes. Some, but not all, k-turns become folded in the presence of metal ions. Whether or not a given k-turn is folded under these conditions is determined by its sequence. We present a set of rules for the prediction of folding properties and the structure adopted on local sequence.

## Highlights

• k-turns are widespread elements that kink the axis of duplex RNA.
• k-turns mediate tertiary contacts and bind proteins.
• Both the folding process and the structure adopted depend on local RNA sequence.
• We have deduced a number of rules to associate sequence and folding.
• The rules have strong predictive power.

## Graphical Abstract

**Figure:**
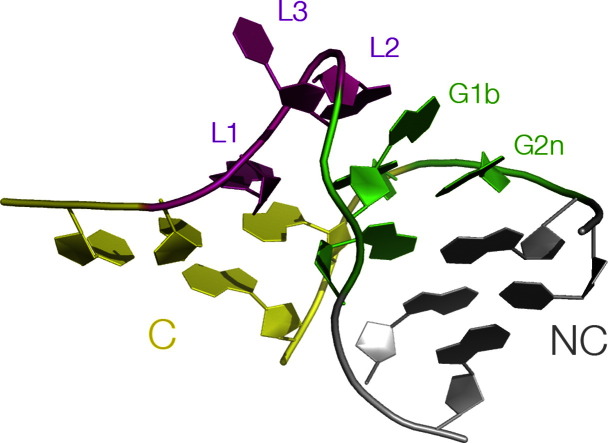

Secondary structure depictions of medium length to long RNA species are generally rather complex, with extensive basepairing and tertiary interactions. However, secondary structure can be reduced to a series of semi-rigid duplex segments connected by helical junctions of different kinds that determine the relative trajectories of the helices and that facilitate long-range tertiary contacts. Thus, it is really the junctions that generate the architecture of the molecules. If we understand the folding properties of the junctions, then we can go a long way to understand the overall fold of the complete RNA molecule. This applies to relatively small, autonomously folding molecules such as riboswitches up to long RNA species such as rRNA, and it is to be expected that long non-coding RNAs will be similar.

In the last decade, one helical junction has emerged as both important and widespread throughout functional RNAs; this is the kink turn, generally abbreviated as k-turn. k-turns are found in double-stranded RNA, most often comprising a three-nucleotide bulge followed on the 3′ side by successive G·A and A·G pairs (Fig. 1). The duplex axis is kinked about the loop, minor groove-to-minor groove, to include an angle close to 50° [1], thus forming a very tight kink in the duplex RNA (Fig. 2a). k-turns were noted as a repeated sequence [2] or kinked structure [3] but were first explicitly described as a new motif within the ribosome by Klein *et al*. [4]. k-turns facilitate tertiary interactions in RNA, often in conjunction with another helical junction. Frequently, one of the component helices is a relatively short stem–loop, the loop of which interacts with some kind of receptor at a remote location in the RNA. For example, most of the k-turns found in the ribosome make such contacts. In the SAM-I riboswitch, a relatively long RNA helix is kinked by a standard k-turn such that the terminal loop can dock into a receptor to create the ligand binding site [5]. k-turns can also serve as specific binding sites for proteins.

**Fig. 1:**
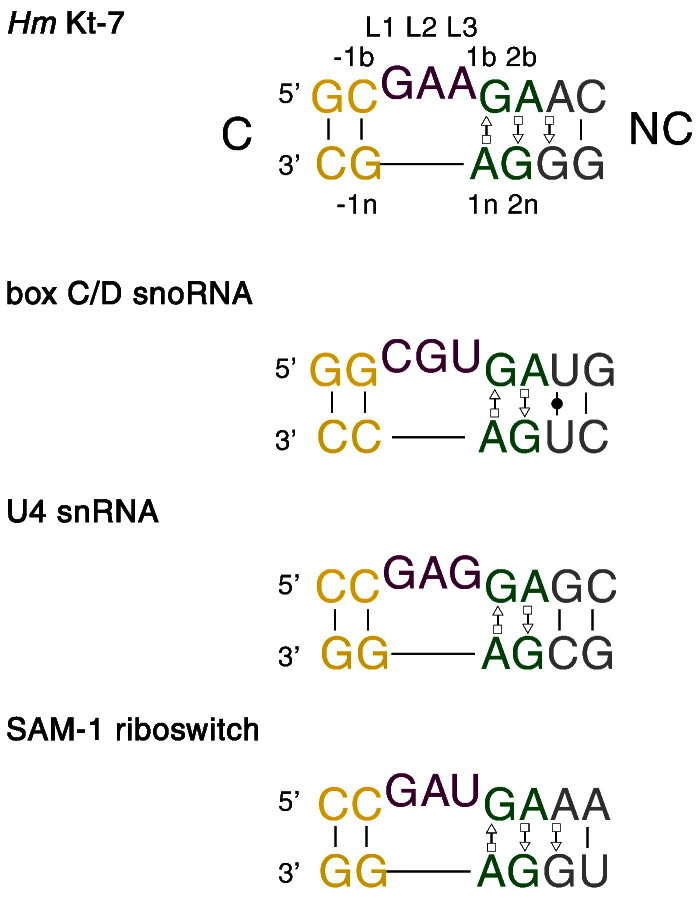
The sequence of standard, simple k-turns. The top shows the sequence of HmKt-7, with the nomenclature of the nucleotide positions indicated. The same coloring is used throughout this review, including the molecular graphics images. The sequences of three more common k-turns are shown.

**Fig. 2:**
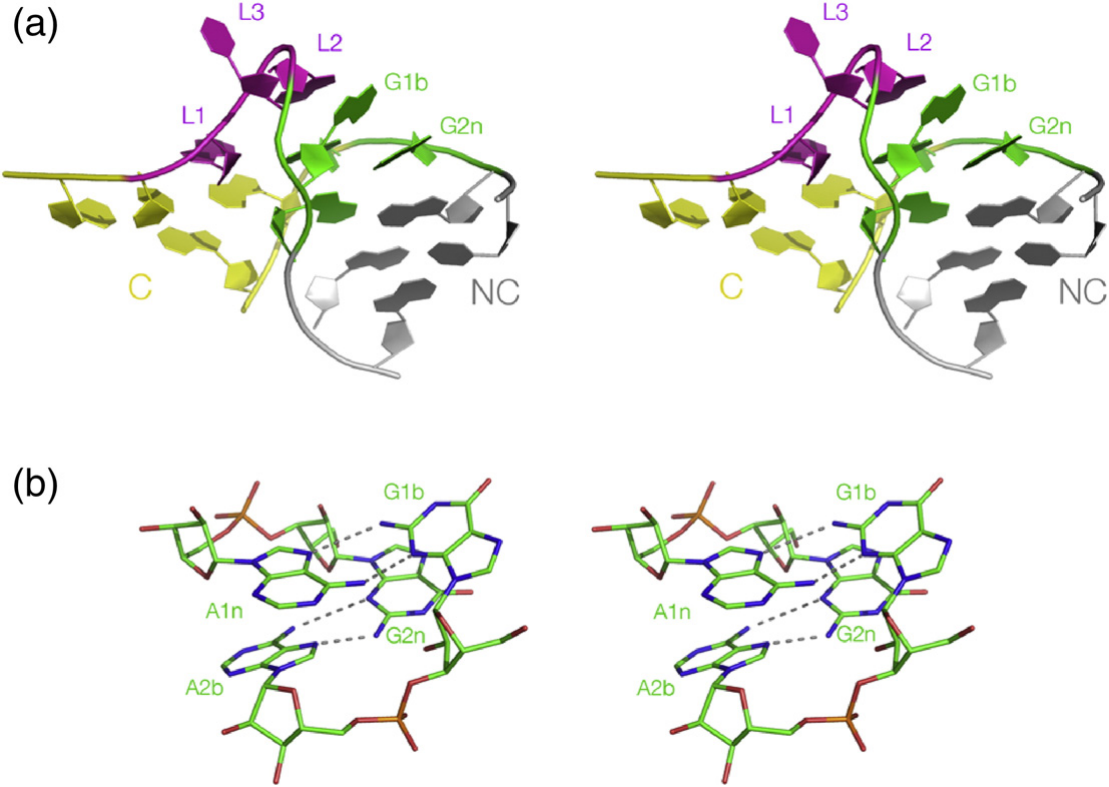
The overall structure of the k-turn, and the G·A pairs at the core. Parallel-eye stereoscopic images are shown. (a) The k-turn structure shown in cartoon form. (b) The two *trans* sugar-Hoogsteen G·A pairs. Note that the minor groove edges of the adenine bases are oriented leftward in this view, that is, are directed toward the minor groove of the C helix.

The structure and folding of k-turns has been extensively analyzed to the point that this is probably the best understood RNA structural motif. The goal in these studies is now to relate the properties of k-turns to their sequence and thus to generate a set of rules to predict the structure and folding from a knowledge of the sequence alone.

## Occurrence of k-turns

While not quite universal, k-turns are extremely widespread in RNA structures, with examples in most classes of functional RNA. There are many different k-turns found throughout ribosomal RNA from bacteria [6], archaea [4], [7], [8] and eukaryotes [9]. The ribosomal k-turn Kt-7 of the archaeon *Haloarcula marismortui* has been especially well studied, and we shall subsequently refer to this as HmKt-7. k-turns play a key organizational role in the assembly of the box C/D and H/ACA snoRNP structures that carry out guided methylation and pseudouridylation of archaeal and eukaryotic RNAs [10], [11], [12], [13], as well as in the U3 snoRNP involved in nucleolytic processing rRNA [14], [15], [16]. The U4 snRNA in the B-complex of the spliceosome cycle that precedes the tri-snRNP complex has a k-turn [3], [17]. k-turns occur in the human signal recognition particle [18] and ribonuclease P [19], while seven different classes of riboswitch [5], [20], [21], [22], [23], [24], [25] have known or putative k-turns. It is therefore apparent that k-turns play a role in many aspects of RNA function, including the translation (ribosome) and modification (snoRNA) of RNA, spliceosome assembly (U4) and the control of gene expression (riboswitches, etc.).

## Nomenclature of k-turns

At its simplest (but see the section on k-turn classification), the k-turn comprises a duplex RNA containing a bulge of most often three nucleotides, followed by G·A and A·G pairs immediately to its 3′ side (Fig. 1 top). The helix to the 5′ side of the bulge is normally Watson–Crick basepaired and is called the C (for **C**anonical) helix, while that containing the G·A pairs is called the NC (for **N**on-**C**anonical) helix. We label the loop nucleotides L1, L2 and so on sequentially from the 5′ end and the remaining nucleotides are given positive numbers in the 3′ direction through the NC helix and negative numbers in the 5′ direction through the C helix [26]. Those on the **b**ulged strand have a suffix b and those on the **n**on-bulged strand have a suffix n.

## The Structure of the k-turn

The heart of the k-turn is the two G·A pairs at the 1b,1n and 2n,2b positions. Both basepairs are *trans* G(sugar edge)·A(Hoogsteen edge) connected by hydrogen bonds from GN2 to AN7 and from AN6 to GN3 (Fig. 2b), although the latter bond is not present in the 2n,2b pair in one class of k-turns (see below). The 1b,1n pair is strongly buckled, with G1b rotated ~ 25° out of plane, whereas the 2n,2b pair is almost planar. The hydrogen bonds of the G·A pairs contribute to the stability of the folded k-turn; ion-induced folding is prevented by removing the guanine exocyclic amino group from either guanine [27]. Both the C and NC helices are capped by stacked nucleobases from the loop. L1 (the 5′ nucleotide of the loop) stacks onto the end of the C helix, while L2 is stacked onto the end of the NC helix, adopting a *syn* conformation that maximizes stacking on A1n. By contrast, L3 is directed into the solvent and makes no contact with the k-turn RNA.

**Fig. 3:**
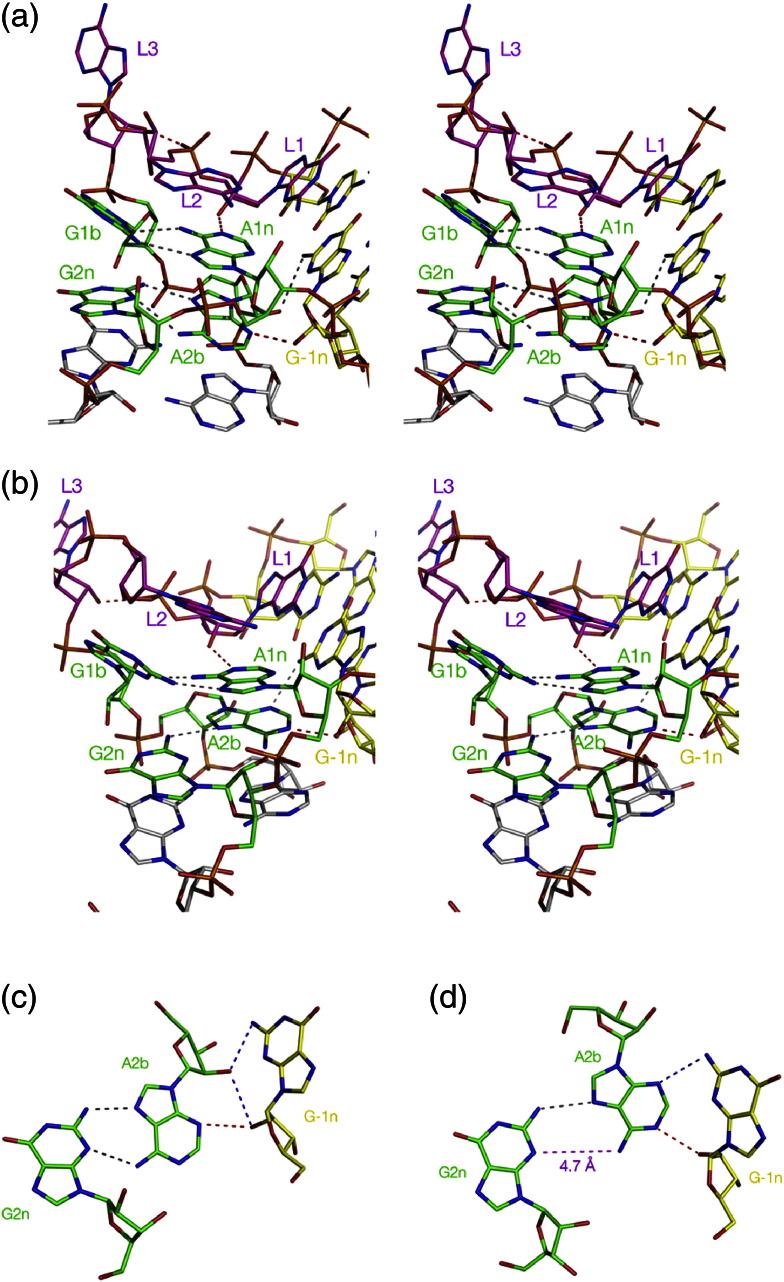
The A-minor interactions and the N3 and N1 structures of k-turns. Hydrogen bonds are shown as broken lines, with the key cross-strand bonds highlighted in red. Parallel-eye stereoscopic images are shown in (a) and (b), viewed from the side of the non-bulged strand. (a) The core structure of HmKt-7 as a free RNA duplex [29] where it adopts a typical N3 structure. (b) The core structure of HmKt-7 *in situ* in the *H*. *marismortui* ribosome [7] where it forms an N1 structure. (c and d) Triple interaction between − 1n and the 2b,2n basepair in typical N3 and N1 structures, as non-stereoscopic views. (c) Taken from the human U4 snRNA k-turn [3] (an N3 structure) while (d) is from HmKt-38 [7] (an N1 structure). Additional hydrogen bonds involving G-1n are shown in blue. In (d), the distance A2b N6 to G2n N3 (shown as magenta broken line) is 4.7 Å, that is, too long to be hydrogen bonded.

The kinked structure of the k-turn juxtaposes the minor grooves of the C and NC helices, allowing a number of near-universal A-minor interactions [28] to stabilize the folded structure. The minor groove edges of the adenine bases of the two G·A pairs (i.e., A1n and A2b) are directed toward the minor groove of the C helix where they accept hydrogen bonds. Two hydrogen bonds are essentially common to all k-turns and are important to the structure (Fig. 3). The first is donated by the L1 O2′ and accepted by A1n N1, that is, the conserved adenine in the G·A pair closest to the bulge. Removal of the O2′ atom from HmKt-7 totally prevented Mg^2 +^-ion-induced folding [26]. The O2′ of the ribose in the − 1n position (in the C helix) donates the second key hydrogen bond, accepted by a ring N of the conserved adenine at the 2b position. The acceptor can be either N3 or N1, discussed in the following section.

Additional hydrogen bonds are found in different k-turns, and some formed adventitiously as permitted by the sequence. In many k-turns, a hydrogen bond is donated by L3 O2′ to the *pro*S non-bridging O of the phosphate linking L1 and L2 to close the neck of the loop. Removal of L3 O2′ in HmKt-7 led to an impairment of ion-induced folding [26], though to a lesser extent than disruption of the L1 to A1n hydrogen bond.

## Two Classes of k-turn Structure

The O2′ of the ribose in the − 1n position can be accepted by A2b either at N3 or at N1 (Fig. 3), and the natural k-turns can be divided on this basis into two classes with about equal numbers in each (Table 1). The change in hydrogen bonding requires a different rotational setting of the A2b nucleobase that affects its basepairing with G2n. The GN2-to-AN7 hydrogen bond is always present, and when the proton from the − 1n O2′ is accepted by A2b N3, then the AN6-to-GN3 bond is also formed. However, acceptance of the hydrogen bond by A2b N1 results in a rotation of the A2b nucleobase that is incompatible with the AN6-to-GN3 bond so that this distance is usually > 4 Å in the N1 class structures (Fig. 3). The overall pattern of hydrogen bonding in the minor groove between A2b and the − 1n nucleotide is different in the two classes. In the N3 structure, the − 1n O2′ can also be hydrogen bonded with the A2b O2′, and where − 1n is guanine (most common), its N2 can also be bonded to A2b O2′. In the N1 structures, the O2′ atoms of 2b and − 1n are too far apart to interact, but the − 1n N2 can donate a proton to A2b N3. In some N1 structures (e.g., the k-turn of the cobalamine riboswitch [24]), there is an O2′-to-O2′ hydrogen bond between the − 2n and 3b ribose, but this interaction is not universal in all the N1 class k-turns.

**Table 1:** Hydrogen bond lengths in a range of standard and non-standard k-turns, showing the division into the N3 and N1 class conformations.

	− 1n to 2b		2b,2n pair		
	O2′ N3	O2′ N1	GN2 N7	AN6 N3	PDB
Box C/D	2.8		2.9	3.1	1RLG
U4 snRNA	3.2		2.7	3.1	1E7K
Kt-46 *H*.*ma*.	2.7		2.8	3.7	1FFK
Cyc diGMP rsw	2.8		2.7	3.0	3Q3Z
SAM rsw	2.6		2.5	3.4	3GX5
SAM + YbxF	3.1		3.0	3.3	3V7E
SAM G2nA	2.8				2YGH
Kt-38 *H*.*ma*.		2.8	2.9	4.7	1FFK
Kt-7 *H*.*ma*.		2.7	3.0	4.3	1FFK
Kt-23 *T*.*th*.		2.6			2WH1
Kt-23 *T*.*so*.		2.5		5.0	4AEB
L30e		2.7	2.7	4.6	1T0K
Kt-11 *T*.*th*.		3.1		4.3	2WH1
cobalamine rsw		2.5	3.1	5.3	4GXY

The top group are N3 structures (mean − 1n O2′ to A2b N3 = 2.9 Å) and the bottom group are N1 structures (mean − 1n O2′ to A2b N1 = 2.8 Å). Note that, in the N1 structures, the mean length of the G2n N3–A2b N6 separation is 4.7 Å, that is, too long to be hydrogen bonded. *H*.*ma*., *H*. *marismortui*; *T*.*th*., *T*. *thermophilus*; *T*.*so*., *T*. *solenopsae*; rsw, riboswitch.

Some k-turns can adopt either N3 or N1 structure depending on their environment. HmKt-7 adopts an N1 class structure in the 50S ribosomal subunit [7]. By contrast, the same Kt-7 sequence placed in the SAM-I riboswitch [1] or as a simple duplex RNA bound to L7Ae or free of protein [29] adopts an N3 class structure in each case. The preferred structure is clearly N3 class, yet something about the environment of HmKt-7 in the ribosome forces it to adopt an N1 structure. We have found similar behavior for Kt-23 of *Thelohania solenopsae*. As a free duplex bound by L7Ae protein, it adopts an N3 structure [30], whereas in the SAM-I riboswitch, it forms an N1 structure [31]. A dynamic equilibrium could exist in free solution, with interconversion between the N3 and N1 class conformations. While there are no experimental data in support of this at present, molecular dynamics trajectories suggest that such transient changes in hydrogen bonding pattern might be possible [32].

The switch between N3 and N1 structures involves more than a local change in hydrogen bonding; it changes the whole shape of the k-turn structure. The distance between the O2′ atoms at the − 2n and 3b positions of HmKt-7 increases by more than 4 Å in changing from the N1 to the N3 conformation. The relative disposition of the C and NC helices is significantly altered by the change in structure. The conformational change was analyzed in terms of three rotation angles relating the position of the C helix relative to the NC helix [1]. These were the axial bend angle (α), the direction of the bend (β) and the rotation around the C helix axis (γ). Analysis of the known k-turn structures revealed a systematic difference in γ between the two conformations. In N3 structures, γ < 50°, while γ > 50° for the N1 class structures. This could clearly influence any tertiary interactions made by the arms of the k-turn.

## Classification of k-turn Structure

**Fig. 4:**
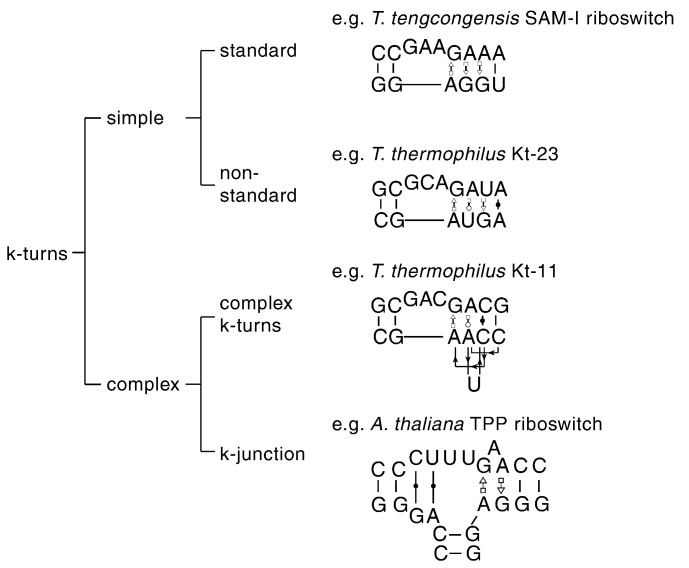
k-turn classification, with examples. k-turns may be divided between simple and complex. Non-standard k-turns have deviations from the standard sequence in the core. The k-junctions have a third helix fused into the non-bulged strand opposite the bulge.

The simple, standard k-turn is formed from two strands as a bulged duplex, with the standard G·A and A·G pairs at the 1b,1n and 2b,2n positions. Examples of such k-turns include HmKt-7 of 23S rRNA, the U4 snRNA, box C/D snoRNA and *Thermoanaerobacter tengcongensis* SAM-I riboswitch k-turns. Simple k-turns subdivide into standard and non-standard classes (Fig. 4). In non-standard simple k-turns, there is a substitution in one of the G·A pairs, most often at the 2b,2n position. Kt-23 of the 16S rRNA provides a good example. The frequency of occurrence of the nucleotide in the 2n position (G in the standard k-turn) is U > C > G > A in different species. Although a G2nU substitution prevents ion-induced folding of Kt-7, Kt-23 of *Thermus thermophilus* (where 2n = U) is well folded under the same conditions [31], [33]. Kt-23 of *T*. *solenopsae* is a rare example of a k-turn with an adenine at the 2n position, that is, where 2b,2n is an A·A pair, which is isosteric with the G·A pair [34]. We determined the structure of this k-turn located in the SAM-I riboswitch [31] where it forms an N1 structure. By contrast, when we modified the natural SAM-I riboswitch k-turn with a G2nA substitution, it forms an N3 class structure [35].

In complex k-turns, the nucleotides of the G·A pairs do not map linearly onto the sequence of the RNA. Despite this, the RNA adopts a normal k-turn structure, with the standard hydrogen bonding features. For this reason, we apply our conventional nomenclature [26], assigning nucleotides according to their position in the three-dimensional structure, rather than the primary sequence. Kt-11 of *T*. *thermophilus* provides an example. In this k-turn, the non-bulged strand of the NC helix reverses direction to form an S-turn so that the 1n and 2n nucleotides are separated by two nucleotides in the primary sequence including a cytosine at the 3n position. Despite this, the A2b is located at its normal position within the structure, participating in the usual A-minor hydrogen bonding of the k-turn.

The k-junction is another class, where the k-turn has been elaborated into a helical junction [36]. Using a computer algorithm to search for the general shape of the k-turn, we found three-way junctions in the structures of the TPP riboswitches of *Escherichia coli* and the plant *Arabidopsis thaliana*. The k-junction exhibits all the key features of a k-turn. The third helix of the junction is coaxially aligned with the C helix, while the k-turn loop forms the turn into the NC helix. Analysis of more than 10,000 TPP riboswitch sequences suggested that they all have this feature. Evidently, the k-turn is structurally well disposed to be incorporated into a three-way helical junction.

## Functional Alternatives to the k-turn Structure

Although the major function of the k-turn is to kink the axis of RNA to enable tertiary interactions to form, it may not be a unique solution to the problem. A k-turn in a particular RNA within one organism may be functionally replaced by an alternative structure in another. Studying a series of lysine riboswitches, Blouin and Lafontaine identified a probable k-turn-forming sequence in the aptamer domain that should facilitate tertiary interaction [21]. They furthermore showed that the *Bacillus subtilis* riboswitch bound the L7Ae protein. However, when the structure of the lysine riboswitch of *Thermotoga maritima* was elucidated [37], [38], the k-turn was not present. Instead, its place was occupied by a different kinked structure lacking G·A pairs and was not stabilized by long-range hydrogen bonds. Another example is provided by ribonuclease P. In this ribozyme, a loop-receptor interaction that creates the binding site for the substrate is facilitated by a tightly kinked RNA helix [39]. In some cases, this is achieved by a k-turn, while in others, a different element called the pk-turn accomplishes the same aim. The *T*. *maritima* pk-turn is globally very similar to a k-turn but lacks the standard features of a k-turn. These two elements achieve the same structural result, and they can functionally substitute for each other. Thus, a SAM-I riboswitch in which the k-turn has been replaced by the pk-turn retains the ability to bind SAM ligand, while *T*. *maritima* RNase P in which the pk-turn is replaced by a k-turn has ribozyme activity [40].

## k-turns as Protein Binding Sites

Many k-turns, including most of the ribosomal k-turns, are bound by specific proteins in their cellular context. As a class, this includes proteins of very different sequence and structure that interact with different faces of the k-turn. However, the L7Ae family of proteins represents the archetypal k-turn binding proteins. These include the eukaryotic and archaeal proteins L7Ae, L30e and S12e [41]; the yeast Nhp2 and Snu13p proteins; and the human 15.5-kDa protein [42]. In addition, there are bacterial homologs that include YbxF [43]. Assembly of boxes C/D and H/ACA nucleoproteins is initiated by the binding of L7Ae-type proteins to k-turns formed in the guide RNA [44], [45], followed by the association of other proteins in an ordered process that culminates with the association of the enzyme that carries out the chemical modification. In addition, L7Ae is a component of the U3 snoRNP [16]. A box H/ACA motif is also found within human telomerase RNA [46]. The 15.5-kDa protein binds the k-turn that forms in the U4 stem–loop of the U4-U6·U5 tri-snRNP [3], [42]. Lastly, L7Ae has been shown to be a subunit of archaeal RNase P [47].

**Fig. 5:**
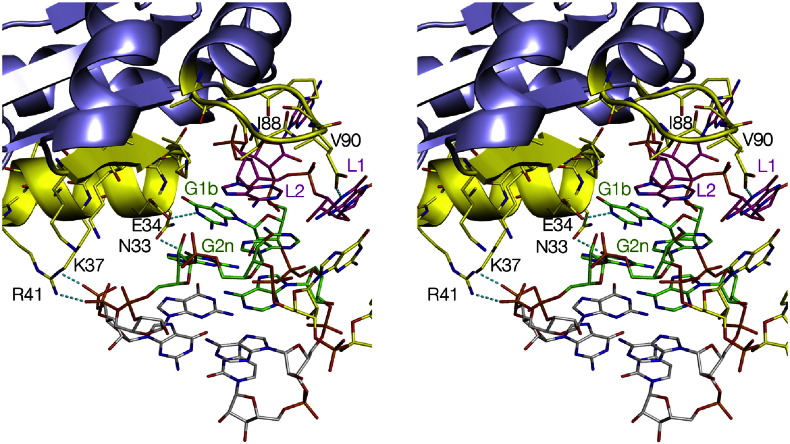
The interaction of the L7Ae protein with HmKt-7 [29]. A parallel-eye stereoscopic image is shown. The bulk of the protein is colored blue, with the key regions that interact with the k-turn highlighted in yellow, including key amino acid side chains shown in stick form. The regions of L7Ae that interact with the k-turn comprise the α-helix whose N-terminus makes specific interactions with the conserved guanine bases and a hydrophobic loop that forms a cap over the loop region of the k-turn. These interactions are general for the L7Ae family of proteins binding to k-turn RNA.

The structure of L7Ae bound to Kt-15 was determined in the context of the *H*. *marismortui* large ribosomal subunit [7]. Crystal structures have also been determined for L7Ae bound to a box C/D k-turn [10] and for human 15.5-kDa protein bound to the U4 snRNA k-turn [3]. The structure of *Archaeoglobus fulgidus* L7Ae bound to HmKt-7 [29] was solved at 2.3 Å resolution (Fig. 5) from which the general principles of recognition were deduced. On folding, the k-turn juxtaposes the minor grooves of the C and NC helices and therefore opens up the major groove on the outside face of the RNA. In normal helical RNA structure, the major groove is both narrow and deep, making it inaccessible, but in the k-turn, it becomes very accessible, and the major groove edges of the conserved guanine bases of the G·A pairs are exposed. These are followed by the L2 and L1 bases before continuing into the C helix. The L7Ae proteins bind in this opened-out major groove, contacting the key elements of the k-turn using two sections of protein. The first is a highly basic β-strand:turn:α-helix element, and the second is a short loop of hydrophobic residues.

The α-helix of the first element is located in the major groove of the NC helix (Fig. 5) in a manner that is strongly reminiscent of the recognition helices of helix–turn–helix proteins binding to their DNA targets. The C-terminus of the helix lies close to the non-bulge-containing strand of the RNA where a number of basic side chains make non-specific interactions with phosphate groups. In addition, two amino acids at the N-terminal end of the β-sheet section direct basic side chains toward the non-bulge strand, contributing to the electrostatic stabilization of the complex. The N-terminal end of the α-helix mediates specific recognition of the k-turn via both guanine bases of the conserved G·A pairs. In all the L7Ae family complexes, the O6 atom of G1b is located at the N-terminal end of the α-helix, almost on its axis. Thus, the partial negative charge on this oxygen atom will be stabilized by the positive pole of the helix dipole. In addition, there are specific hydrogen bonds formed with the guanine bases. The glutamate side chain of E34 in the first turn is hydrogen bonded to N1 of G1b; this interaction seems to be universal for the protein family. The adjacent N33 asparagine is hydrogen bonded to O6 of G2n; this is also found in the box C/D interaction.

The second element of the L7Ae protein recognizes the L2 and L1 nucleobases, essentially covering them with a hydrophobic loop that buries a surface area of 732 Å^2^ (Fig. 5). Isoleucine I88 is located directly over L2 in the complex, the *syn* conformation of which maximizes its contact with the loop.

Taken together, these elements generate a very specific molecular recognition of the structure of the k-turn in double-stranded RNA. By placing the α-helix in the major groove of the NC arm and capping the L2 nucleobase, it effectively “measures” the angle between these two elements in the major groove. This then juxtaposes the N-terminus of the α-helix with the major groove side of the guanine bases of the two conserved G·A where side chains make specific hydrogen bonds, and G1b O6 is placed to maximize the interaction with the positive pole of the helix dipole.

## The Folding of k-turns

An RNA duplex containing a k-turn-forming sequence can exist as a tightly kinked k-turn structure or a more extended conformation typical of any three-base bulge. Measurements of fluorescence lifetime indicate that, in the absence of added metal ions or binding proteins, an RNA duplex containing the HmKt-7 sequence exists in a conformational equilibrium between these two forms, biased toward the extended structure [48], [49]. We have identified three ways in which the population of folded k-turn species can be increased.

Some k-turns adopt the kinked geometry on addition of metal ions. This can be easily studied either as a retardation of mobility in gel electrophoresis or by the increase in the efficiency of fluorescence resonance energy transfer between fluorophores attached to the 5′ termini of the C and NC helices resulting from the shortening of the end-to-end distance [48]. HmKt-7 undergoes folding on addition of divalent or monovalent cations, in a two-state process with [Mg^2 +^]1/2 = 80 μM or [Na^+^]1/2 = 30 mM and a Hill coefficient *n* ~ 1 [26]. However, not all k-turns fold in the presence of metal ions alone. The sequence determinants of these differences are discussed in the following section.

As noted previously, most k-turns are involved in tertiary interactions, and these can also lead to stabilization of the kinked structure. This was demonstrated using the *T*. *tengcongensis* SAM-I riboswitch where a standard k-turn kinks a long helix to help generate the ligand binding pocket. Thus, folding could be studied indirectly by the heat evolved on ligand binding using isothermal calorimetry [35]. The key role of the k-turn was shown by making an A1nC substitution, leading to a failure to bind SAM. Making a G2nA substitution (creating an A·A pair at the 2b,2n position) prevented k-turn folding as an isolated duplex on addition of metal ions. However, the same sequence located in the riboswitch allowed normal binding of SAM and solving the structure of the modified riboswitch by X-ray crystallography showed it to be folded normally (RMSD = 0.53 Å) despite the presence of the substitution. Thus, the very same sequence was unable to fold into the k-turn conformation as a free duplex but nevertheless underwent folding when incorporated into the complete riboswitch. From this, we conclude that the impaired k-turn adopts the kinked structure when its free energy of folding is coupled to that of the riboswitch via the tertiary interaction.

The third process that leads to stabilization of k-turn conformation is the binding of proteins. Binding of L7Ae to HmKt-7 results in folding into the kinked conformation in the absence of metal ions [50], and binding of other ribosomal proteins, L24 and S11, similarly result in the folding of their cognate k-turns (L.H. & D.M.J.L., unpublished results). The affinity of binding can be very high; we have measured *K*_d_ = 10 pM for L7Ae binding to HmKt-7 [27]. In principle, the formation of k-turn structure on binding might occur by passive selection of the kinked structure or a more active process in which the protein manipulates the RNA structure. This was investigated using single-molecule experiments, studying the binding of fluorescently labeled RNA to immobilized L7Ae protein [51]. Real-time binding experiments failed to detect an extended intermediate down to a time resolution of 16 ms. While we could not exclude conformational changes on a faster timescale, the results were consistent with a conformational selection model whereby the protein selects a fraction of RNA that is already in the kinked conformation, thereby drawing the equilibrium into this form.

## Sequence Dependence of k-turn Folding

HmKt-7 folds into the kinked conformation on addition of metal ions. However, not all k-turns can fold in response to metal ions alone. Neither box C/D nor U4 k-turns undergo folding in response to any concentration of metal ions. They are not intrinsically incapable of adopting the k-turn conformation because both fold on binding L7Ae protein, as previously demonstrated crystallographically [3], [10]. Thus, each can adopt the k-turn conformation, yet metal ions alone are insufficient to induce folding. What then is the key difference between k-turns that are folded by metal ions alone and those that are not? Both HmKt-7 and the U4 k-turns are standard k-turns with G·A and A·G pairs at the 1b,1n and 2b,2n positions, respectively, and both have C·G pairs at the − 1b,− 1n position. We therefore examined the role of the 3b,3n position and systematically altered this in HmKt-7 to all the 16 possibilities [52]. We found a range of folding abilities from full folding for natural HmKt-7 (where 3b,3n = A·G) to those exhibiting a complete inability to fold under these conditions (e.g., 3b,3n = G·C). However, all the sequences folded upon binding L7Ae protein; thus, none was intrinsically unable to form the k-turn structure. Thus, the 3b,3n sequence is clearly a key discriminator determining whether or not the k-turn will fold in metal ions alone.

**Fig. 6:**
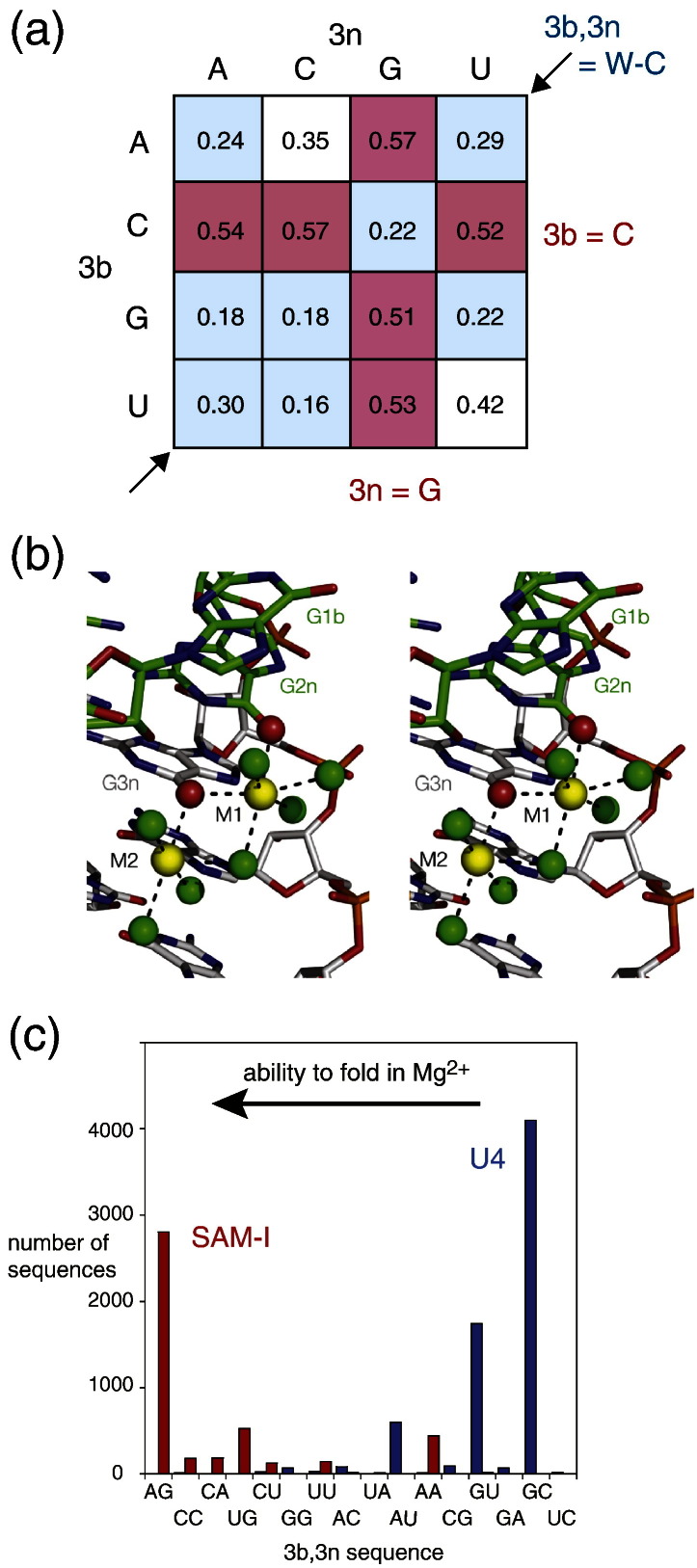
The role of the 3b,3n sequence in the ability of k-turns to be folded on addition of metal ions in the absence of tertiary contacts or protein binding [52]. (a) The folding of 16 HmKt-7 variants with all possible 3b,3n sequences on addition of Mg^2 +^ ions was measured by fluorescence resonance energy transfer. An array of the values of the final fluorescence resonance energy transfer efficiency (*E*_FRET_) as a function of the 3b and 3n sequences is shown. Sequences that are readily folded by Mg^2 +^ ions (*E*_FRET_ ≥ 0.5) are shaded red, while those poorly folded (*E*_FRET_ ≤ 0.3) are shaded blue. The ascending diagonal contains the Watson–Crick pairs. (b) A crystal structure of HmKt-7 showing two hydrated Mg^2 +^ ions (colored yellow) bound in the major groove of the NC helix, shown as parallel-eye stereographic images. Note that the O6 atoms (red spheres) of both G2n and G3n are directly coordinated to the metal ions, displacing inner-sphere water molecules of hydration (green spheres). (c) Correlation of 3b,3n sequence with biological function. Histogram showing the distribution the 3b,3n sequences of the k-turns of SAM-I riboswitches (red) and U4 snRNA species (blue) in natural sequences, ranked (L to R) by the ability to fold in Mg^2 +^ ions. Note that the SAM-I sequences are clustered to the left (able to fold in metal ions), whereas the U4 sequences are clustered to the right (unable to fold in metal ions).

The ability of Kt-7 to undergo ion-induced folding as a function of the 3b,3n sequence is depicted in array form in Fig. 6a. Examination of this suggests four empirical rules governing the folding behavior.

Rule 1. Watson–Crick pairs (ascending diagonal) plus G·U all fold weakly or not at all, with G·C and C·G being especially bad. The U4 k-turn follows this rule.

Rule 2. The presence of 3n = G (third column) confers an ability to fold in Mg^2 +^ ions. Natural HmKt-7 follows this rule.

Rule 3. The presence of 3b = C (second row) confers an ability to fold in Mg^2 +^ ions.

Rule 4. Rule 1 takes precedent over Rules 2 and 3 because 3b,3n = C·G is unfolded in metal ions.

We can understand some of these rules at a molecular level at present. A high-resolution crystal structure of HmKt-7 offers an explanation of Rule 2. A structure of HmKt-7 as a simple duplex was solved at 2.0 Å resolution [52], examination of which revealed two hydrated metal ions bound in the major groove of the NC helix adjacent to G2n and G3n. The ions (probably Mg^2 +^) had clear octahedral symmetry, with inner-sphere ligands that included both water molecules and guanine O6 atoms (Fig. 6b). One ion had exchanged two adjacent inner-sphere water molecules with the O6 atoms of G2n and G3n, while G3n O6 made inner-sphere contacts with both ions. This pattern of coordination lacing up the major groove clearly requires that nucleotide 3n is guanine, as required by Rule 2. Removal of the O6 atom from G3n (i.e., substitution by 2-aminopurine) led to significant impairment of folding in solution [27], [52]. Crystallographic study of RNA duplexes containing a central G·A, A·G step showed that these adopt the *trans* sugar-Hoogsteen basepairs similar to those of the k-turn when flanked by U·G pairs but form *cis* Watson–Crick pairs when flanked by G·C pairs (L.H. & D.M.J.L., unpublished results). This offers a partial explanation of Rule 1.

Our deduced folding rules correlate with biological function. We analyzed the distribution of 3b,3n sequences in several thousand examples of two functional RNA species, the k-turns of the SAM-I riboswitch and U4 snRNA [52]. These two situations represent contrasting environment and function. The SAM-I k-turn mediates a tertiary contact in the riboswitch [5], [35] and is not known to bind a protein, whereas the U4 snRNA k-turn binds the 15.5-kDa protein during spliceosome assembly [42]. If we plot the occurrence of the 3b,3n sequences for the two species ranked by the ion-induced folding ability of modified HmKt-7 with the same 3b,3n sequence, then we find that the SAM-I and U4 species are grouped at opposite ends of the folding scale (Fig. 6c). For the SAM-I k-turns, 60% have 3b,3n = A·G (i.e., the best-folding sequence), and just 0.1% are C·G or G·C (the worst). By contrast, only 0.03% of the U4 k-turn sequences have 3b,3n = A·G, and 97% are predicted to be unable to fold in Mg^2 +^ ions. Mutation of the U4 k-turn sequence to convert 3b,3n from G·C to A·G conferred an ability to fold in response to addition of Mg^2 +^ ions, that is, changing the 3b,3n sequence is sufficient to switch the folding behavior completely. It appears that the SAM-I riboswitch k-turns have been selected for predisposition to fold in metal ions alone. Given that the riboswitch is not known to bind a protein, it must be able to fold unaided to generate a functional structure. The U4 k-turn binds the 15.5-kDa protein *in vivo*; thus, this will remain unfolded until the protein binds. Until this occurs, the k-turn is likely to be more flexible; this may allow the formation of other interactions during the biogenesis of this complex and dynamic assembly that only become fixed on binding the 15.5-kDa protein. Thus, we see that there is a correlation between the folding properties conferred by the 3b,3n sequence and the biological function of specific k-turns.

Application of the folding rules to the k-turns of the *H*. *marismortui* 50S ribosomal subunit [7] is informative. 3b,3n = A·G in Kt-7, Kt-46, Kt-58 and Kt-78, together with the J4,5 k-junction, and these k-turns are therefore likely to fold before protein binding occurs. Examination of many bacterial Kt-7 sequences reveals that 99.9% have either 3n = G or 3b = C, although 3b,3n = A·G is not frequent. In contrast, Kt-15 is a complex k-turn with 3b,3n = C·G that will not fold on addition of metal ions alone (unpublished results). Its folding and subsequent formation of tertiary contacts should therefore occur later than that of the k-turns that do not require protein binding. These differential properties are expected to contribute to an ordered assembly process for the ribosome.

Is this the whole story? Preliminary evidence indicates that other parts of the k-turn, including the − 1b,− 1n sequence and the loop, also have a role to play in determining the folding characteristics in response to metal ions. These studies are ongoing and will hopefully lead to a refinement of the folding rules in due course.

## Sequence Dependence of k-turn Conformation

**Fig. 7:**
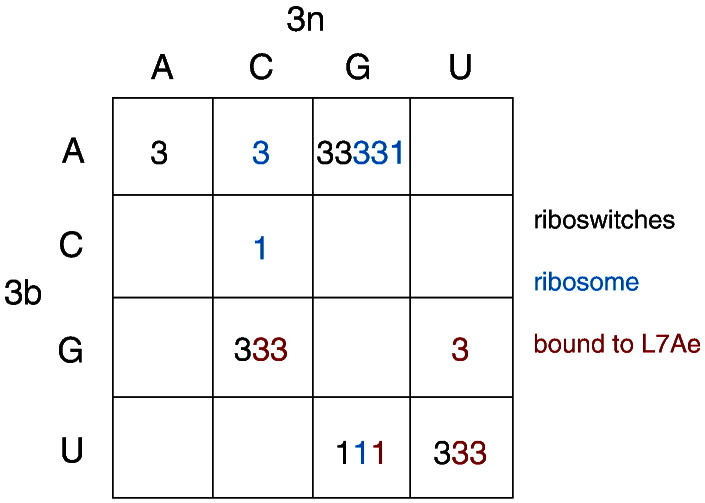
The role of the 3b,3n sequence in determining whether an N3 or an N1 conformation is adopted by a given natural k-turn. The structures of 18 k-turn structures have been analyzed and plotted in array form as a function of 3b,3n sequence. The origin of the k-turns is indicated by color; those in riboswitches are written black, ribosomes are in blue and complexes with L7Ae protein are in red.

In addition to the folding properties, it is also clear that the equilibrium structure of the k-turn must also be sequence dependent. As noted above, some k-turns adopt the N3 structure while others form the N1 structure. Examination of the sequences of 18 natural k-turns of known structure in a variety of environments reveals that, like folding, the conformation also correlates with the 3b,3n sequence. This is depicted in array form in Fig. 7. These different k-turns have 8 of the 16 possible 3b,3n pairings. With one exception (discussed below), a given 3b,3n sequence is associated with a single conformation. Further data from laboratory-designed k-turns expand and, in many cases, confirm these results (L.H. & D.M.J.L., unpublished results). It is clear that 3b,3n = G·C or U·U results in an N3 conformation, whereas 3b,3n = U·G leads to an N1 conformation. Moreover, we see that the latter case applies to k-turns in a riboswitch (cobalamine [24]), in the ribosome (Kt-7 in *T*. *thermophilus*[53]) and in L30e pre-mRNA bound by *Saccharomyces cerevisiae* L30e protein [54]. The empirical rules implied by Fig. 7 allow us to predict the structure of a k-turn, although the molecular basis for these rules is not yet understood. The exception is Kt-7 in the *H*. *marismortui* ribosome, which adopts the N1 structure. However, this has 3b,3n = A·G, which leads to an N3 conformation in every other case. Indeed, in all other circumstances we have examined, Kt-7 adopts the N3 structure, including when it was inserted into the SAM-I riboswitch [1] and as a duplex RNA either free or bound by the L7Ae protein [29]. In the ribosome, Kt-7 is bound by the L24 protein, and its C helix makes a tertiary contact; both of these could exert a conformational influence on the k-turn. However, analysis of many ribosomal sequences reveals that > 99% of natural Kt-7 sequences have an N1-forming 3b,3n pair, and 92% are U·G. Thus, clearly Kt-7 is selected for N1 conformation in the vast majority of cases, and for some unknown reason, the *H*. *marismortui* ribosome is the very rare exception to this. Nevertheless, apart from this single exception, the association between the 3b,3n sequence and the conformation adopted by the k-turn remains good.

## In Conclusion

We see that k-turns play a key conformational role in the formation of medium- and large-scale RNA structures and thus are important in many of the functions of cellular RNA. Probably, the single-most important role of k-turns is to mediate tertiary interactions; very often one or other of the helical arms is involved in a loop-receptor interaction of some kind. Many k-turns are also the targets of specific protein binding. During the biogenesis of functional assemblies such as the ribosome, it is expected that k-turns will initially be relatively flexible, allowing the exploration of conformational space. However, the formation of successful tertiary contacts will begin to reduce this flexibility, and the structure could then be fixed by the binding of protein to the k-turn. Both the folding process and the eventual structure adopted depend on the local nucleotide sequence, especially at the 3b,3n sequence, and these will clearly affect the function. It is therefore extremely important to understand the rules that associate the sequence with the folding and structural properties. A set of rules that have strongly predictive power are beginning to emerge, and it can be applied to new RNA sequences as they emerge.
